# Validation of a frailty phenotype screening questionnaire for rural Chinese older adults: a cross-sectional study

**DOI:** 10.1186/s12877-025-06754-3

**Published:** 2025-12-08

**Authors:** Hui Xie, Jing Gao, Yanfang Zhang, Shuzo Kumagai, Meng Zhao, Ming Li, Si Chen

**Affiliations:** 1 School of Nursing and Rehabilitation, Shandong University, Jinan, 250012 China; 2 Shandong Institute of Commerce and Technology, Jinan, 250103 China; 3 Affiliated Hospital of Jining Medical University, Jining, 272000 China; 4 Kumagai Institute of Health Policy, Fukuoka, 813-0044 Japan; 5 Institute of Digital Health Care, Dong-A University, Busan, 49236 Republic of Korea

**Keywords:** Fried frailty phenotype, Older adults, Reliability, Validity, Rural population

## Abstract

**Background:**

Frailty represents a significant public health issue among older adults in rural China. While the Fried Frailty Phenotype (FFP) is one of the most widely used and well-validated tools for assessing physical frailty, its reliance on physical performance measures and specialized devices limits its feasibility for large-scale screening in such settings. Therefore, a simple and feasible screening instrument based on the FFP framework is needed.

**Aim:**

To validate the Chinese Frailty Phenotype Questionnaire (CFPQ) for rural Chinese older adults.

**Methods:**

This cross-sectional study enrolled 1590 rural-dwelling adults (≥ 65 years) in northern China. The 5-item dichotomous questionnaire operationalized the criteria of the Fried Frailty Phenotype (FFP) endorsed by the Chinese Medical Association (CMA) and included five items: Fatigue, Resistance, Ambulation, Inactivity and Loss of weight. The psychometric evaluation assessed reliability (including internal consistency and test-retest stability), construct validity (including convergent and discriminant validity) and criterion validity. Criterion validity was evaluated through diagnostic accuracy (ROC analysis) and agreement with the criterion standard (kappa statistic).

**Results:**

The CFPQ showed a test-retest intraclass correlation coefficient of 0.89. The internal consistency, measured using Kuder-Richardson formula 20, was 0.49. Construct validity was assessed by calculating correlation coefficients between CFPQ items and cross-sectional outcomes, which ranged from − 0.33 to 0.23. Convergent validity was supported by theoretically consistent correlations: Ambulation was negatively associated with gait speed (*r *= -0.33, *p* < 0.001); Resistance with the 30-second chair stand test (*r *= -0.23, *p* < 0.001); Fatigue with the EQ-5D Visual Analogue Scale (*r *= -0.20, *p* < 0.001), and Loss of weight with body mass index (*r *= -0.11, *p* < 0.001). Discriminant validity was evidenced by statistically distinct patterns of correlations across domains. Against the Fried Frailty Phenotype (FFP-CMA) criterion, the CFPQ achieved an area under the curve of 0.93 (95% CI: 0.92–0.95). A score of ≥ 3 was identified as the optimal cut-off, with a specificity of 97.3%, a negative predictive value of 98.9%, and a positive predictive value of 34.4%. The kappa statistic indicating agreement between the CFPQ and FFP-CMA was 0.41.

**Conclusion:**

The CFPQ demonstrates acceptable validity and reliability as a practical tool for frailty phenotype screening among rural-dwelling older adults in China.

**Clinical trial number:**

Not applicable.

**Supplementary Information:**

The online version contains supplementary material available at 10.1186/s12877-025-06754-3.

## Introduction

Frailty is a clinical state of increased vulnerability to stressors due to age-related decline in physiological reserves, significantly predicting adverse health outcomes including falls, disability, hospitalization, and mortality [1, 2]. Home to the world’s largest and most rapidly aging population, China confronts a critical public health challenge posed by frailty [3]. A recent meta-analysis reported that the prevalence of frailty among adults aged ≥ 60 years in China is 10.1% [4], with even higher rates observed in rural versus urban populations [5]. This disparity may be exacerbated by rural–urban inequalities in socioeconomic status, healthcare access, and nutritional status [6, 7], establishing a clear need for screening and preventive strategies targeting these rural communities.

The Fried Frailty Phenotype (FFP) represents one of the most widely adopted assessment frameworks in both research and clinical settings [8, 9]. However, its implementation in rural China faces practical challenges, as the standard assessment requires objective performance measures (e.g., grip strength, gait speed), dedicated space, and technically trained personnel [9]. These requirements pose barriers in rural areas, where healthcare resources are often limited compared to urban centers [6]. In addition, lower levels of health literacy are well-documented in rural populations, which can hinder the understanding of standardized assessment instructions for complex tools like the FFP [10, 11].

To address these implementation challenges, a frailty phenotype-based screening tool that does not require performance-based tests or specialized equipment is necessary for large-scale frailty identification in rural China. Prior questionnaire versions of the FFP have been developed in Japan and Korea [12, 13]. Notably, the Japanese Fried Frailty Phenotype Questionnaire (FFPQ) and the Korean Frailty Phenotype Questionnaire (FPQ) have replaced objective measures of grip strength and gait speed with self-reported items capturing equivalent functional domains. Furthermore, the calculation-based assessment for physical inactivity has been substituted with direct self-reported measures. These adapted instruments maintain the core construct of the FFP while enhancing feasibility for community-based screening, providing a practical methodological framework. However, a validated instrument tailored for rural older adults in China has not been established.

Therefore, to bridge this gap, the present study aims to validate the Chinese Frailty Phenotype Questionnaire (CFPQ), a pragmatic self-report instrument, specifically for use among rural community-dwelling older adults.

## Methods

### Study design and participants

This cross-sectional study was conducted within the framework of the HALE (Healthy Aging and Lifestyle Enhancement) project. The HALE project is a community-based initiative that collects multidimensional data in rural areas to explore associations between lifestyle, physical activity, and health among older populations. It aims to provide scientific evidence for enhancing health and supporting healthy aging.

The study was carried out from April to August 2024 in a rural township in northern China. During investigator-initiated health screenings provided at no cost through the HALE research protocol, 1,906 adults aged 65 years and above were recruited. Data were collected by assessors with professional backgrounds in geriatric care and rehabilitation, all of whom completed a standardized training program. All participants underwent a comprehensive assessment that included both the direct measurements for the FFP-CMA and the self-reported CFPQ. Exclusion criteria included individuals with acute medical events, terminal illnesses, or severe cognitive impairment. Among the initial 1906 participants who completed the assessments, 316 were excluded due to missing data on frailty and physical performance, leaving 1590 participants for the final analysis. Ethical approval was granted by the Ethics Review Committee at Shandong University in China (Approval No. 2024-R-036). All participants provided informed consent.

### The Fried Frailty Phenotype (FFP-CMA) and Chinese Frailty Phenotype Questionnaire (CFPQ)

#### Fried Frailty Phenotype (FFP-CMA)

The objective frailty assessment was based on the FFP adapted for the Chinese population, endorsed by the Chinese Medical Association(CMA) [14]. It evaluates five components as follows:

### Exhaustion

One point for exhaustion was allocated if participants reported that either of the following conditions occurred on 3 or more days during the past week: (1) “I felt that everything I did was an effort,” or (2) “I could not get going.”

### Grip strength

Grip strength was assessed on the self-reported dominant hand, with the highest of two trials recorded for analysis. A score of 1 was assigned if the value was below the FFP-CMA cutoffs defined by sex and BMI. For males, the criteria were as follows: ≤29 kg for BMI ≤ 24.0 kg/m², ≤ 30 kg for BMI 24.1–26.0 kg/m² or 26.1–28.0 kg/m², and ≤ 32 kg for BMI > 28.0 kg/m². For females, the criteria were ≤ 17 kg for BMI ≤ 23.0 kg/m², ≤ 17.3 kg for BMI 23.1–26.0 kg/m², ≤ 18 kg for BMI 26.1–29.0 kg/m², and ≤ 21 kg for BMI > 29.0 kg/m².

### Walking speed

Walking speed was assessed by measuring the time taken to complete a 4.57-meter walk at a usual pace. A score of 1, indicating slowness, was assigned based on sex- and height-stratified cutoffs. For males, slowness was defined as a time ≥ 7 s for those ≤ 173 cm in height, or ≥ 6 s for those > 173 cm. For females, the criteria were a time ≥ 6 s for height > 159 cm, or ≥ 7 s for height ≤ 159 cm.

### Physical inactivity

For the physical inactivity criterion, a score of 1 was assigned if the participant’s weekly energy expenditure, as assessed by the International Physical Activity Questionnaire-Short Form (IPAQ-SF), fell below the established sex-specific thresholds of 383 kcal for males and 270 kcal for females.

### Weight loss

Participants were assigned a score of 1 for weight loss if they reported an unintentional loss of > 4.5 kg or > 5% of body weight in the past year.

### Frailty score

Participants were classified as frail with a score of ≥ 3, as pre-frail with a score of 1–2, and as robust with a score of 0.

#### Chinese Frailty Phenotype Questionnaire (CFPQ)

Following the framework of the FFP-CMA, the assessment method for exhaustion was retained, while the weight loss criterion was defined as an unintentional weight loss of more than 4.5 kg in the past year. The objective grip strength measurement was substituted with the self-report question: “By yourself and not using aids, can you normally climb 10 stairs?” The 4.57-meter walk test was replaced by an item evaluating aerobic endurance: “By yourself and not using aids, do you have any difficulty walking 1 km without resting?” Finally, the energy expenditure criterion was replaced by the self-report item: “Does your sitting or lying time account for 80% or more of your waking time in a day?” (Table 1).

**Table 1:** English version of the CFPQ

Item	Questions	Answer Options	Score
Fatigue	Choose option 3 or option 4 on either of these two questions from the CES-D: How many days did the following situations occur in the past week? (1) I felt everything I did was an effort. (2) I could not get going.	option 1: < 1 d option 2: 1–2 d option 3: 3–4 d option 4: > 4 d	1 = option 3 or 4 0 = option 1 or 2
Resistance	By yourself and not using aids, can you normally climb 10 stairs?	option 1: No; option 2: Yes	1 = option 1 0 = option 2
Ambulation	By yourself and not using aids, do you have any difficulty walking 1 km without resting?	option 1: No; option 2: Yes	1 = option 2 0 = option 1
Inactivity	Does your sitting or lying time account for 80% or more of your waking time in a day?	option 1: No; option 2: Yes	1 = option 2 0 = option 1
Loss of weight	Have you unintentionally lost more than 4.5 kg of weight in the past year?	option 1: No; oprion 2: Yes	1 = option 2 0 = option 1

*BMI *Body mass index, *CES-D* the Center for Epidemiologic Studies Depression, *CFPQ *Chinese Frailty Phenotype Questionnaire

The stair-climbing item was selected based on its validation in established Asian frailty phenotype questionnaires, including the Japanese FFPQ [12] and the Korean FPQ [13], as both grip strength and stair-climbing evaluate muscular strength—a domain that frailty instruments should be designed to capture [15]. The ambulation item is consistent with the Japanese FFPQ. While similarly, the Korean FPQ uses a 400-meter walk for its assessment. Furthermore, the ability to walk 1 km is also recognized as an indicator of ambulation within the Mini Sarcopenia Risk Assessment (MSRA) [16]. For the inactivity item, the adopted threshold of ≥ 80% of waking time spent on sedentary aligns with that of the Japanese FFPQ. This consistency is further supported by evidence that older individuals with low physical activity spend, on average, 74.0% of their waking hours in sedentary behavior. A study found that older adults who spent approximately 70% of their waking time in sedentary behavior at baseline had an equal presence and absence of frailty 2 years later [17]. Furthermore, the same study reported a positive association between the percentage of sedentary behavior and frailty risk, with each 10% increase corresponding to a 55% higher risk (HR = 1.55). Therefore, a threshold of 80% was selected to define physical inactivity in the present study.

### Cross-sectional outcomes

A series of cross-sectional outcomes were assessed. Body mass index (BMI) was measured using a bioelectrical impedance analyzer (Model MC-980MA; TANITA, JPN). Grip strength was assessed with an electronic hand dynamometer (Model EH101; Camry, CHN). Physical performance measures included: (1) gait speed measured over a 5-meter distance; (2) one-leg standing test with eyes open; (3) 30-second chair stand test, recording the number of stands completed; (4) the five-repetition sit-to-stand test (5STS), with the completion time recorded in seconds. Total physical activity was objectively measured using triaxial accelerometers (Model wGT3X-BT; ActiGraph, USA). Vital capacity was assessed using an electronic spirometer (Model EFH-100B; Tiyue, CHN). Finally, health-related quality of life (HRQoL) was self-reported using the European Quality of Life 5 Dimensions Visual Analogue Scale (EQ5D-VAS) [18].

### Statistical analysis

All the statistical analyses were performed with Stata 18.0. The data are presented as mean ± standard deviation (SD) for continuous variables and frequency (percentage) for categorical variables. The psychometric reliability of the instrument was evaluated as follows: internal consistency reliability was evaluated using the Kuder‒Richardson Formula 20 (KR-20), with a value ≥ 0.7 considered acceptable [19]. Test-retest reliability was assessed using the intraclass correlation coefficient (ICC) based on a two-way mixed-effects model for absolute agreement over a 14-day interval (*n* = 60). ICC values below 0.40 indicated poor reliability and values of 0.75 and above indicated excellent reliability [20]. Construct validity was assessed by examining convergent validity with theoretically related outcomes and discriminant validity with theoretically unrelated outcomes. These correlations were calculated as point-biserial correlations (for normally distributed outcomes), or Spearman’s rho correlations (for non-normal outcomes), based on the distribution of the data. All five items are included for validation to operationalize the core domains of the FFP-CMA. Criterion validity was assessed against the FFP-CMA. The diagnostic accuracy, evaluated by indicators such as sensitivity and specificity, was evaluated across all possible cut-off points, and receiver operating characteristic (ROC) curves were analyzed to determine the area under the curve (AUC) with 95% confidence intervals. The kappa values were assessed to indicate the agreement between the CFPQ and FFP-CMA: 0.41–0.60 for moderate agreement and 0.61–0.80 for substantial agreement [21].

## Results

### Participant characteristics

The baseline characteristics of the sample are shown in Table 2. The participants had an average age of 72.61 years (SD = 4.32), while 48.93% of the participants were male. According to the FFP-CMA, 877 participants (55.16%) were categorized as robust, 674 (42.39%) as prefrail, and 39 (2.45%) as frail.

**Table 2:** Characteristics of the overall sample

Characteristics	(Mean ± SD) or *n* (%)
Age, year	72.61 ± 4.32
Gender, male	778(48.93)
BMI, kg/m^2^	24.53 ± 3.61
Grip strength, kg	26.38 ± 7.66
Gait speed, m/s	1.03 ± 0.21
One-leg standing ^a^	2.76 ± 2.73
Chair stand test (30-second) ^b^	13.82 ± 3.95
Time to complete 5 chair rises ^c^	11.63 ± 3.57
Vital capacity ^d^	2206.92 ± 715.81
Total Physical Activity Time, min/d ^e^	368.24 ± 135.87
EQ5D-VAS score ^f^	79.69 ± 16.03
EEPA, kcal/week	4614.64 ± 2659.14
Fried Frailty Phenotype	
Robust	877(55.16)
Prefrail	674(42.39)
Frail	39(2.45)

*BMI* Body mass index, *EQ5D-VAS* European Quality of Life 5 Dimensions Visual Analogue, *EEPA* Energy expenditure of physical activity

^e^ Missing values: *n* = 103; ^f^ Missing values: *n* = 4 *n* = 1590; ^a^ Missing values: *n* = 58; ^b^ Missing values: *n* = 66; ^c^ Missing values: *n* = 67; ^d^ Missing values: *n* = 5

### Reliability

The reliability analysis of the CFPQ indicated a KR-20 of 0.49 for internal consistency and an ICC of 0.89 for test-retest reliability.

### Construct validity

Table 3 presents the correlation coefficients for each item in relation to cross-sectional outcomes. Convergent validity was supported by significant correlations between components and their theoretically related measures. Specifically, weight loss was negatively associated with BMI. Fatigue was negatively correlated with EQ5D-VAS scores. Resistance was negatively associated with grip strength, one-leg standing time, and the 30-second chair stand test. Ambulation was associated with slower gait speed. Discriminant validity was evidenced by differential patterns of correlations across domains with cross-sectional outcomes.

**Table 3:** Correlation coefficients and *P*-values between CFPQ items and Cross-Sectional outcomes

Cross-sectional Outcomes	Fatigue		Resistance		Ambulation		Inactivity		Loss of weight	
	Correlation	*P* Value	Correlation	*P* Value	Correlation	*P* Value	Correlation	*P* Value	Correlation	*P* Value
BMI	0.05	0.06	0.12	< 0.001	0.13	< 0.001	−0.04	0.10	−0.11	< 0.001
Grip strength	−0.10	< 0.001	−0.17	< 0.001	−0.17	< 0.001	0.03	0.25	−0.0007	0.98
Gait speed	−0.21	< 0.001	−0.29	< 0.001	−0.33	< 0.001	−0.04	0.09	−0.06	0.01
One-leg standing ^a^	−0.07	0.007	−0.11	< 0.001	−0.13	< 0.001	−0.007	0.78	−0.004	0.87
Chair stand test (30-second) ^b^	−0.17	< 0.001	−0.23	< 0.001	−0.26	< 0.001	0.03	0.31	0.02	0.38
Time to complete 5 chair rises ^c^	0.15	< 0.001	0.21	< 0.001	0.23	< 0.001	−0.02	0.47	−0.02	0.48
Vital capacity ^d^	−0.10	< 0.001	−0.18	< 0.001	−0.20	< 0.001	0.01	0.62	0.03	0.21
Total Physical Activity Time ^e^	−0.11	< 0.001	−0.12	< 0.001	−0.14	< 0.001	−0.04	0.11	−0.02	0.38
EQ5D-VAS ^f^	−0.20	< 0.001	−0.22	< 0.001	−0.23	< 0.001	−0.05	0.07	−0.06	0.02

Spearman’s correlation coefficients were reported, as all continuous variables deviated from normality (Shapiro-Wilk test, *P* < 0.05)

*BMI *Body mass index,* EQ5D-VAS *European Quality of Life 5 Dimensions Visual Analogue Scale

^a^ Missing values: *n* = 58; ^b^ Missing values: *n* = 66; ^c^ Missing values: *n* = 67; ^d^ Missing values: *n* = 5; ^e^ Missing values: *n* = 103; ^f^ Missing values: *n* = 4

### Criterion validity

**Fig. 1:**
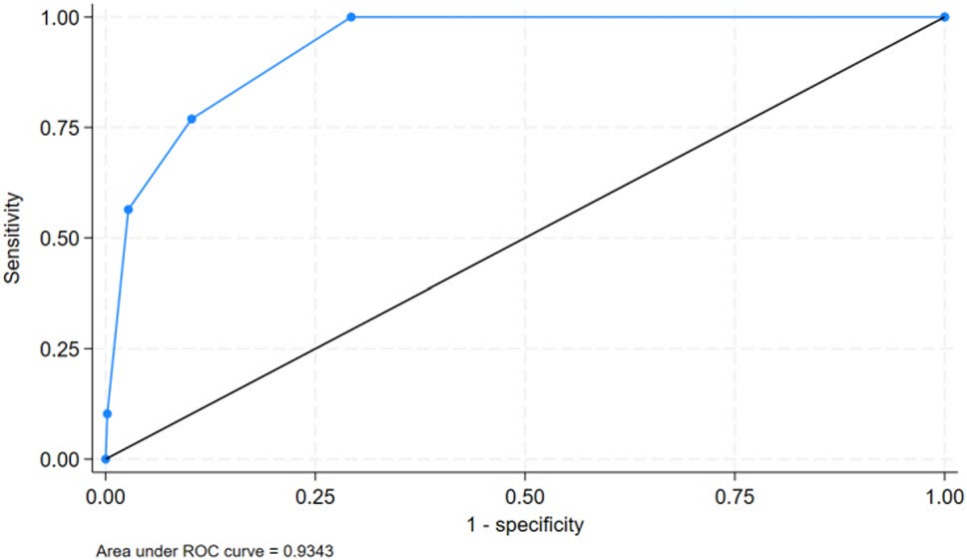
ROC Curve of the CFPQ for Frailty Phenotype Detection Using the FFP-CMA as the Criterion Standard

The diagnostic accuracy of the CFPQ is summarized in Table 4. The high AUC value (0.93, 95% CI: 0.92–0.95) (Fig. 1) indicates that the CFPQ effectively discriminates frail and non-frail participants. Compared with a threshold of 3 (Table 4), a threshold of 2 demonstrated higher sensitivity (76.9% vs. 56.4%) and identified a larger proportion of individuals with the frailty phenotype. However, this lower threshold was associated with reduced specificity (89.8% vs. 97.3%), a lower positive predictive value (15.9% vs. 34.4%), and lower diagnostic agreement with the FFP-CMA (Kappa = 0.23 vs. 0.41). Furthermore, the two instruments demonstrated comparable effect sizes (Cohen’s d) when discriminating frail from non-frail groups on adverse health outcomes (Supplementary Table 1).

**Table 4:** Diagnostic accuracy of the CFPQ using the FFP-CMA as the criterion standard

Cut-off	Frail, *n*(%)	AUC (95%CI)	Youden Index	Sensitivity (%)	Specificity (%)	PPV (%)	NPV (%)	Kappa
Cut-off ≥ 2	189(11.9)	0.93(95%CI: 0.92–0.95)	0.67	76.9	89.8	15.9	99.4	0.23*
Cut-off ≥ 3	64(4.0)		0.54	56.4	97.3	34.4	98.9	0.41*

*P < 0.001

## Discussion

This study validates the CFPQ, the first frailty phenotype-based screening tool developed for community-dwelling older adults in rural China. The CFPQ demonstrated high test–retest reliability (ICC > 0.75) and excellent discrimination of frailty status, as evidenced by an AUC of 0.93 against the FFP-CMA criterion [22]. The optimal cut-off score of ≥ 3 yielded high specificity (97.3%) and negative predictive value (98.9%), supporting its utility as a practical screening tool in resource-limited settings.

The limited internal consistency observed aligns with previous studies and reflects the heterogeneous nature of frailty domains [9, 12, 23]. Unlike cumulative deficit models that aggregate functionally homogeneous deficits, the CFPQ assesses distinct physiological domains (e.g., fatigue, resistance, ambulation), which are influenced by different biological mechanisms and correlate with distinct clinical outcomes [9, 24]. Moreover, this instrument demonstrated higher test-retest reliability (ICC = 0.89) than the Japanese FFPQ (ICC = 0.72) [12].

Construct validity was evidenced by domain-specific associations between CFPQ items and cross-sectional outcomes. The correlation between objective grip strength and the self-reported resistance item was −0.17 (*p* < 0.001), which reflects the distinction between measured upper-limb strength and self-perceived lower-limb function. However, it showed a higher correlation with the 30-second chair stand test (*r* = −0.23), a performance-based measure of lower-limb strength, providing evidence for its convergent validity. Additionally, the observed moderate correlation (*r* = −0.33) between the ambulation item and the gait speed measure, despite capturing distinct constructs (self-perceived aerobic capacity versus short-distance velocity), is consistent with the established physiological link whereby declining aerobic capacity necessitates a reduction in walking speed [25].

The observed lack of a significant association between self-reported inactivity and accelerometer-measured physical activity aligns with established evidence of limited concordance between self-report and device-based assessments [26, 27]. This discrepancy is consistently demonstrated by studies showing non-significant correlations and poor agreement across diverse populations and tools [28, 29]. Furthermore, a systematic review found that single-item self-report measures generally underestimate sedentary time compared to device-based measurements [30]. This systematic underestimation may be more pronounced in the current low-education sample (mean schooling: 3.58 ± 3.41 years), as evidence indicates that self-reported physical activity bias is greater among individuals with lower educational attainment, who tend to overestimate their daily physical activity [31, 32].

The CFPQ exhibited higher discriminatory accuracy than several widely used frailty instruments such as the Japanese FFPQ (AUC = 0.88) [12] and the Korean FPQ (AUC = 0.89) [13]. Furthermore, it achieved high specificity (97.3%) and a high negative predictive value (98.9%), indicating that in practice, the tool can effectively identify individuals at low risk for frailty to enable healthcare providers to prioritize further assessment and resource allocation for the remaining higher-risk subgroup. The criterion validity was further supported by comparable effect sizes (Cohen’s d) observed for groups classified by the CFPQ and the FFP-CMA across multiple health indicators (Supplementary Table 1).

### Strengths and limitations

The CFPQ is the first frailty phenotype-based questionnaire validated for rural-dwelling older adults in China. Its simple design enables self-reporting without physical measurements and allows a single interviewer to complete the assessment in under two minutes using paper-based forms in homes, rural health stations, or by telephone. These features support its feasibility for large-scale screening in resource-limited settings with minimal infrastructure or specialized personnel.

This study has limitations. First, the sample was recruited from a single Chinese city, which may limit its generalizability. Second, the cross-sectional design precludes the longitudinal evaluation of the questionnaire’s capacity to predict adverse outcomes. A further limitation concerns the cognitive demand of certain fatigue-related items. The phrasing “I could not get going” may pose interpretation challenges for some older adults, potentially compromising the validity of responses to these specific items without additional interviewer clarification.

## Conclusions

The CFPQ is a valid screening instrument for identifying frailty phenotype in rural areas of China for older adults. Its ease of application renders it a practical tool for screening frailty phenotype in this specific population.

## Supplementary Information

Supplementary Material 1

## Data Availability

The data that support the findings of this study are available from the corresponding author upon reasonable request.

## Abbreviations

AUC: Area Under the Curve
BMI: Body Mass Index
CFPQ: Chinese Frailty Phenotype Questionnaire
CMA: Chinese Medical Association
FFP: Fried Frailty Phenotype
HALE: Healthy Aging & Lifestyle Enhancement
ICC: Intraclass Correlation Coefficient
KR-20: Kuder‒Richardson Formula 20
NPV: Negative Predictive Value
PPV: Positive Predictive Value
HRQoL: Health-Related Quality of Life
EQ5D-VAS: European Quality of Life 5 Dimensions Visual Analogue Scale

## References

[1] Hoogendijk EO, Afilalo J, Ensrud KE, Kowal P, Onder G, Fried LP. Frailty: implications for clinical practice and public health. Lancet. 2019;394(10206):1365–75. 31609228 10.1016/S0140-6736(19)31786-6

[2] Clegg A, Young J, Iliffe S, Rikkert MO, Rockwood K. Frailty in elderly people. Lancet. 2013;381(9868):752–62. 23395245 10.1016/S0140-6736(12)62167-9 PMC4098658

[3] Chen X, Giles J, Yao Y, Yip W, Meng Q, Berkman L, et al. The path to healthy ageing in China: a Peking University-Lancet commission. Lancet. 2022;400(10367):1967–2006. 36423650 10.1016/S0140-6736(22)01546-X PMC9801271

[4] Zhou Q, Li Y, Gao Q, Yuan H, Sun L, Xi H, et al. Prevalence of frailty among Chinese community-dwelling older adults: a systematic review and meta-analysis. Int J Public Health. 2023;68:1605964. 37588041 10.3389/ijph.2023.1605964 PMC10425593

[5] Zeng XZ, Meng LB, Li YY, Jia N, Shi J, Zhang C, et al. Prevalence and factors associated with frailty and pre-frailty in the older adults in China: a national cross-sectional study. Front Public Health. 2023;11:1110648. 37554734 10.3389/fpubh.2023.1110648 PMC10406229

[6] Chau KL. Ecological analysis of health care utilisation for China’s rural population: association with a rural county’s socioeconomic characteristics. BMC Public Health. 2010;10:664. 21044343 10.1186/1471-2458-10-664 PMC2988739

[7] Han J, Wu MC, Yang T. Challenge of China’s rural health. BMJ. 2016;353:i2003. 27072539 10.1136/bmj.i2003

[8] Buta BJ, Walston JD, Godino JG, Park M, Kalyani RR, Xue QL, et al. Frailty assessment instruments: systematic characterization of the uses and contexts of highly-cited instruments. Ageing Res Rev. 2016;26:53–61. 26674984 10.1016/j.arr.2015.12.003 PMC4806795

[9] Fried LP, Tangen CM, Walston J, Newman AB, Hirsch C, Gottdiener J, et al. Frailty in older adults: evidence for a phenotype. J Gerontol Biol Sci Med Sci. 2001;56(3):M146–156. 10.1093/gerona/56.3.m146 11253156

[10] Li Y, Lv X, Liang J, Dong H, Chen C. The development and progress of health literacy in China. Front Public Health. 2022;10:1034907. 36419995 10.3389/fpubh.2022.1034907 PMC9676454

[11] Aljassim N, Ostini R. Health literacy in rural and urban populations: a systematic review. Patient Educ Couns. 2020;103(10):2142–54. 32601042 10.1016/j.pec.2020.06.007

[12] Chen S, Chen T, Kishimoto H, Susaki Y, Kumagai S. Development of a fried frailty phenotype questionnaire for use in screening community-dwelling older adults. J Am Med Dir Assoc. 2020;21(2):272-e2761. 31522878 10.1016/j.jamda.2019.07.015

[13] Kim S, Kim M, Jung HW, Won CW. Development of a frailty phenotype questionnaire for use in screening community-dwelling older adults. J Am Med Dir Assoc. 2020;21(5):660–4. 31672563 10.1016/j.jamda.2019.08.028

[14] Hao Q, Li J, Dong B. Chinese Experts Consensus on Assessment and Intervention for Elderly Patients with Frailty. Chin J Geriatr. 2017;36:251–56. 10.3760/cma.j.issn.0254-9026.2017.03.007.

[15] de Vries NM, Staal JB, van Ravensberg CD, Hobbelen JSM, Olde Rikkert MGM. Nijhuis-van der Sanden MWG. Outcome instruments to measure frailty: a systematic review. Ageing Res Rev. 2011;10(1):104–14. 20850567 10.1016/j.arr.2010.09.001

[16] Rossi AP, Micciolo R, Rubele S, Fantin F, Caliari C, Zoico E, et al. Assessing the risk of sarcopenia in the elderly: the mini sarcopenia risk assessment (MSRA) questionnaire. J Nutr Health Aging. 2017;21(6):743–9.28537342 10.1007/s12603-017-0921-4

[17] Song J, Lindquist LA, Chang RW, Semanik PA, Ehrlich-Jones LS, Lee J, et al. Sedentary behavior as a risk factor for physical frailty independent of moderate activity: results from the osteoarthritis initiative. Am J Public Health. 2015;105(7):1439–45. 25973826 10.2105/AJPH.2014.302540 PMC4463377

[18] Rabin R, de Charro F. EQ-5D: a measure of health status from the EuroQol group. Ann Med. 2001;33(5):337–43. 10.3109/07853890109002087 11491192

[19] Streiner DL. Starting at the Beginning: An Introduction to Coefficient Alpha and Internal Consistency. JPers Assess. 2003;80:99–103. 10.1207/S15327752JPA8001_18. 10.1207/S15327752JPA8001_18 12584072

[20] Cicchetti DV, Sparrow SA. Developing criteria for establishing interrater reliability of specific items: applications to assessment of adaptive behavior. Am J Ment Defic. 1981;86(2):127–37. 7315877

[21] Landis JR, Koch GG. The measurement of observer agreement for categorical data. Biometrics. 1977;33(1):159–74. 843571

[22] Hosmer D.W, Lemeshow S, Sturdivant R.X. Assessing the Fit of the Model. In Applied Logistic Regression. 3rd ed. Lnc: New Jersey. Wiley; 2013. pp. 173–182. 10.1002/9781118548387.ch5.

[23] Dong L, Qiao X, Tian X, Liu N, Jin Y, Si H, et al. Cross-cultural adaptation and validation of the FRAIL scale in Chinese community-dwelling older adults. J Am Med Dir Assoc. 2018;19(1):12–7. 28757330 10.1016/j.jamda.2017.06.011

[24] Aprahamian I, Lin SM, Suemoto CK, Apolinario D, Oiring de Castro Cezar N, Elmadjian SM et al. Feasibility and Factor Structure of the FRAIL Scale in Older Adults. J Am Med Dir Assoc. 2017;18(4):367.e11-367.e18. 10.1016/j.jamda.2016.12.067 28214239

[25] Fiser WM, Hays NP, Rogers SC, Kajkenova O, Williams AE, Evans CM, et al. Energetics of walking in elderly people: factors related to gait speed. J Gerontol Biol Sci Med Sci. 2010;65(12):1332–7. 10.1093/gerona/glq137 20679072

[26] Skender S, Ose J, Chang-Claude J, Paskow M, Brühmann B, Siegel EM, et al. Accelerometry and physical activity questionnaires - a systematic review. BMC Public Health. 2016;16:515. 27306667 10.1186/s12889-016-3172-0 PMC4910242

[27] Vetter VM, Özince DD, Kiselev J, Düzel S, Demuth I. Self-reported and accelerometer-based assessment of physical activity in older adults: results from the Berlin Aging Study II. Sci Rep. 2023;13(1):10047. 37344489 10.1038/s41598-023-36924-5 PMC10284919

[28] Husu P, Vähä-Ypyä H, Tokola K, Sievänen H, Rocha P, Vasankari T. Reliability and validity of self-reported questionnaires assessing physical activity and sedentary behavior in Finland. Int J Environ Res Public Health. 2024;21(6):686. 38928933 10.3390/ijerph21060686 PMC11203568

[29] Arumugam A, Alsaafin N, Shalash RJ, Qadah RM, Al-Sharman A, Moustafa IM, et al. Concurrent validity between self-reported international physical activity questionnaire short form and Fibion accelerometer data among young adults in the UAE. Eur J Med Res. 2024;29(1):426. 39155363 10.1186/s40001-024-01975-5 PMC11331689

[30] Prince SA, Cardilli L, Reed JL, Saunders TJ, Kite C, Douillette K, et al. A comparison of self-reported and device measured sedentary behaviour in adults: a systematic review and meta-analysis. Int J Behav Nutr Phys Act. 2020;17(1):31. 32131845 10.1186/s12966-020-00938-3 PMC7055033

[31] Winckers ANE, Mackenbach JD, Compernolle S, Nicolaou M, van der Ploeg HP, De Bourdeaudhuij I, et al. Educational differences in the validity of self-reported physical activity. BMC Public Health. 2015;15:1299. 26856811 10.1186/s12889-015-2656-7 PMC4746888

[32] Lagerros YT, Mucci LA, Bellocco R, Nyrén O, Bälter O, Bälter KA. Validity and reliability of self-reported total energy expenditure using a novel instrument. Eur J Epidemiol. 2006;21(3):227–36. 16547838 10.1007/s10654-006-0013-y

