# Supplementary material for: Validation of a frailty phenotype screening questionnaire for rural Chinese older adults: a cross-sectional study
Source: BMC Geriatr. 2025 Dec 8;26:45. doi: 10.1186/s12877-025-06754-3 (PMC12797398; doi:10.1186/s12877-025-06754-3)
Supplement: Supplementary file 1 — Supplementary Material 1 [file 12877_2025_6754_MOESM1_ESM.docx]

Supplementary Table 1 Comparison of Cross-sectional Outcomes Between Frail and Non-Frail Groups Stratified by FFP-CMA and CFPQ

| Cross-sectional Outcomes | FFP-CMA | | | | CFPQ | | | | Δ \|d\| |
| --- | --- | --- | --- | --- | --- | --- | --- | --- | --- |
|  | Non-Frailty (n=1551) | Frailty (n=39) | P Value | Cohen's d | Non-Frailty (n=1526) | Frailty (n=64) | P Value | Cohen's d |  |
| BMI | 24.51± 3.59 | 25.49±4.57 | 0.18 | -0.27(95%CI:-0.59~0.04) | 24.46±3.57 | 26.25±4.29 | 0.001 | -0.50(95%CI:-0.75~-0.25) | 0.23 |
| Grip strength | 26.53±7.63 | 20.54±6.76 | <0.001 | 0.79(95%CI:0.47~1.11) | 26.57±7.58 | 21.82±8.12 | <0.001 | 0.62(95%CI:0.37~0.88) | 0.17 |
| Gait speed | 1.04±0.19 | 0.64±0.24 | <0.001 | 2.02(95%CI:1.70~2.35) | 1.04±0.20 | 0.76±0.21 | <0.001 | 1.44(95%CI:1.18~1.69) | 0.58 |
| One-leg standing ^a^ | 2.77±2.74 | 1.74±1.13 | <0.001 | 0.38(95%CI: -0.01~0.77) | 2.79±2.75 | 1.77±1.48 | <0.001 | 0.37(95%CI:0.09~0.66) | 0.01 |
| Chair stand test(30-second) ^b^ | 13.90±3.92 | 9.32±2.67 | <0.001 | 1.17(95%CI:0.80~1.55) | 13.92±3.92 | 10.71±3.50 | <0.001 | 0.82(95%CI:0.53~1.11) | 0.35 |
| Time to complete 5 chair rises ^c^ | 11.54±3.46 | 16.71±5.13 | <0.001 | -1.48(95%CI:-1.86~-1.10) | 11.51±3.43 | 15.26±5.24 | <0.001 | -1.07(95%CI:-1.36~-0.78) | -0.41 |
| Vital capacity ^d^ | 2216.50±716.11 | 1827.26±597.30 | <0.001 | 0.55(95%CI:0.23~0.86) | 2228.53±709.59 | 1693.42±674.73 | <0.001 | 0.76(95%CI:0.50~1.01) | -0.21 |
| Total Physical  Activity Time ^e^ | 371.64±134.47 | 238.53±126.63 | <0.001 | 0.99(95%CI:0.67~1.32) | 372.21±135.24 | 276.89±117.79 | <0.001 | 0.71(95%CI:0.45~0.96) | 0.28 |
| EQ5D-VAS ^f^ | 80.10±15.66 | 62.76±21.52 | <0.001 | 1.10(95%CI:0.77~1.42) | 80.49±15.19 | 60.55±22.54 | <0.001 | 1.28(95%CI:1.03~1.54) | -0.18 |

Note: BMI, body mass index; EQ5D-VAS, European Quality of Life 5 Dimensions Visual Analogue Scale; Δ |d | = d _FFP_ – d _CFPQ;_ FFP-CMA: Fried Frailty Phenotype recommended by the Chinese Medical Association; CFPQ: Chinese Frailty Phenotype Questionnaire; ^a^ Missing Values = 58; ^b^ Missing Values = 66; ^c^ Missing Values = 67; ^d^ Missing Values = 5; ^e^ Missing Values = 103; ^f^ Missing Values = 4
